# Successful Treatment of Malignant Priapism by Radiotherapy: Report of a Case, Review of the Literature, and Treatment Recommendations

**DOI:** 10.7759/cureus.17287

**Published:** 2021-08-18

**Authors:** Daniel T Xing, Helene Yilmaz, Supan Hettige, Rajendra Hegde, Rohan Nair

**Affiliations:** 1 Oliver Newton-John Cancer Wellness and Research Centre, Austin Health, Melbourne, AUS; 2 Pathology, TissuPath, Melbourne, AUS; 3 Gippsland Radiation Oncology, Latrobe Regional Hospital, Traralgon, AUS; 4 Radiation Oncology, William Buckland Radiotherapy Centre, The Alfred Health, Melbourne, AUS

**Keywords:** priapism, prostate cancer, radiotherapy, palliative radiation therapy, volumetric modulated arc therapy

## Abstract

Malignant priapism is a condition of painful induration and erection of the penis secondary to metastatic infiltration by a neoplasm. This condition is associated with a poor prognosis. We report on a case of an 87-year-old man who presented with a painful, partially erected penis subsequent to a diagnosis of metastatic Gleason 4+5 prostate cancer. Magnetic resonance imaging (MRI) showed diffuse bilateral infiltration of his corpora cavernosa. The core biopsy of the penile nodule revealed it to be a poorly differentiated carcinoma consistent with prostatic origin. The patient’s symptoms were completely resolved after treatment with high-dose palliative conformal radiotherapy (40Gy in 16 fractions). We systemically reviewed clinical reports of palliative radiotherapy for malignant priapism with the aim to gain more information on the management of this rare condition.

## Introduction

Penile metastasis is a rare phenomenon, first described in 1870 by Eberth. Since then, about 460 cases have been reported in the literature [1-3]. The most common causes of this condition are genitourinary (especially prostate and bladder primaries) and recto-sigmoid primary malignancies [4]. About 20-30% of patients with penile metastasis reported in the literature presented with priapism [5-6]. Malignant priapism is a condition of painful induration and erection of the penis secondary to metastatic infiltration by a neoplasm. Here, we describe a case of malignant priapism secondary to prostate adenocarcinoma. The patient achieved long-lasting symptomatic relief after receiving high-dose palliative radiotherapy to the penis.

## Case presentation

An 87-year-old man with metastatic prostate adenocarcinoma presented with painful and persistent partially erected penis He was previously diagnosed with prostate cancer in 1998 with a prostate-specific antigen (PSA) of 70 µg/L and T3 disease. It was unclear if he had metastatic disease at the time of diagnosis, The patient had been continuously treated with hormonal therapy since his initial diagnosis of prostate cancer. Upon presentation, the patient had low volume bony disease on the seventh cervical spine, first thoracic spine, left femur, and right tibia on a whole-body bone scan. We instituted treatment with leuprorelin and bicalutamide. The patient’s PSA was reduced to 0.75 µg/L.

**Figure 1:**
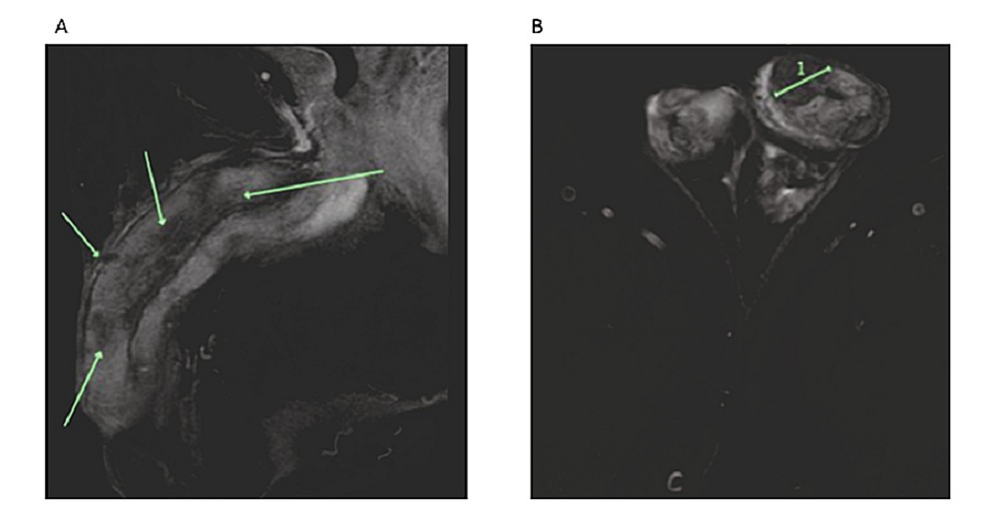
Penile MRI scan of the patient A) Sagittal view of T1 fat suppression sequence with contrast. Arrow: reduced contrast uptake. B) Axial view of T2 fat suppression sequence, 1 Length: 1.92 cm

Figure 1 shows the magnetic resonance imaging (MRI) scan.

MRI showed the heterogeneous signal intensity of the bilateral corpora cavernosa with enhancement. There were poorly defined areas with reduced contrast uptake. There also appeared to be nodules on the right aspect of the glans of the penis and corpus cavernosum involving the tunica albuginea.

**Figure 2:**
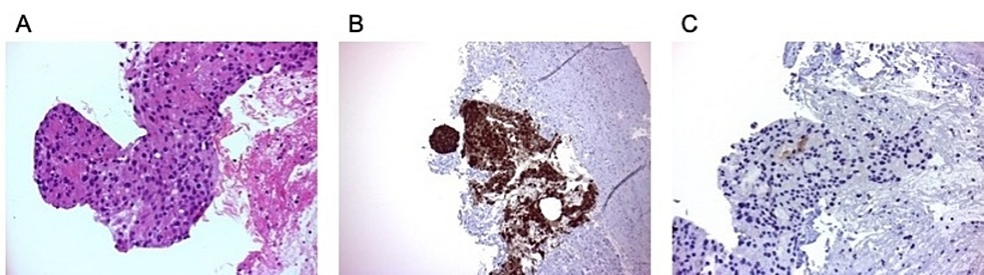
Histological examination of the core biopsy of the penile mass from the patient A) Medium power view showing poorly differentiated carcinoma on hematoxylin and eosin-stained section. B) Poorly differentiated carcinoma highlighted with CK20 stain. C) Poorly differentiated carcinoma with focal staining for prostate-specific membrane antigen (PSMA).

Histological examination of the core biopsy of the penile mass revealed a poorly differentiated carcinoma comprising epithelioid tumor cells with nested and sheet-like architecture, nuclear pleomorphism, prominent nucleoli, and scattered mitotic figures (Figure 2, panel A).

Immunohistochemistry showed tumor cells with positive staining for CK7 and CK20 (Figure 2, panel B), as well as very focal positive staining for PSMA (Figure 2, panel C). GATA3, CK5/6, p63, and PSA stains returned negative. Whilst the presence of staining for CK7 and CK20 is unusual for prostatic adenocarcinoma, a previous prostatic biopsy had shown a similar immunohistochemical staining profile (i.e. with positive staining for CK7, CK20, PSMA, PSAP, and focal staining for PSA); hence, the penile mass biopsy was favored to be metastatic prostatic adenocarcinoma.

**Figure 3:**
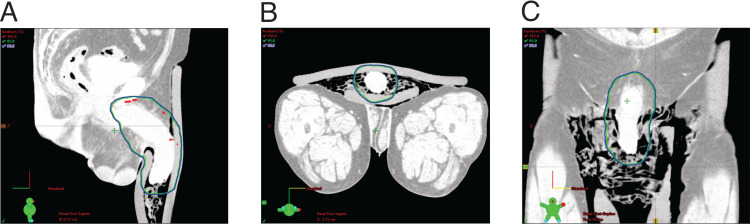
Radiotherapy dosimetry plan for the patient A) Sagittal view. B) Transverse view. C) Coronal view. Blue: 90% isodose line; Green: 95% isodose line; Red: 105% isodose line

The patient had previously received high-dose palliative radiotherapy to the prostate primary (60Gy in 30 fractions). The patient remained to have good performance status. Therefore, we chose to use volumetric modulated arc therapy (VMAT) to deliver moderately hypofractionated high-dose radiotherapy to the penis with the aim to reduce damage to normal pelvic tissue. The patient was treated in a supine position with a 1 cm bolus administered to the penis. The air gap was filled with wet gauze. We initially planned to deliver doses up to 50Gy in 20 fractions. The radiation dosimetry is shown in Figure 3.

**Figure 4:**
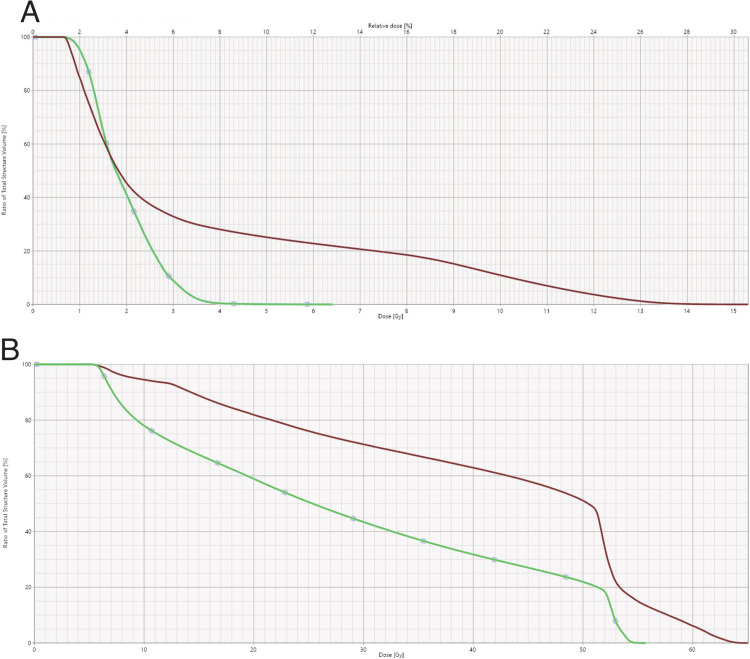
Dose-volume histogram (DVH) of bladder and rectum A) The current course of radiotherapy. B) Combined DVH of the current plan with previous pelvic radiotherapy.

The dose-volume histogram (DVH) of the current plan is shown in Figure 4, panel A.

The bladder and rectum received a very low dose wash. The combined DVH of the bladder and rectum from two courses of pelvic radiotherapy is shown in Figure 4, panel B. Both the bladder and rectum met the dose constraints as per our departmental policy. Daily cone-beam CTs were used for treatment setup verification. However, the patient developed a grade 3 skin reaction of the penis and thus treatment was ceased at 40Gy.

The patient achieved complete symptomatic relief and acute skin toxicity was resolved at two months post-radiotherapy. The patient was switched to androgen depression therapy with abiraterone. At nine months post-radiotherapy, his PSA remains at a low level (0.13 µg/L). He remains symptom-free and maintains excellent urinary function. He further reports a good quality of life at nine months post-radiotherapy.

## Discussion

We carried out a structured literature search regarding malignant priapism and its treatment. The search was limited to English language literature available on the MEDLINE database and to case reports occurring between January 1940 and December 2018. The search terms included priapism, penile, metastasis, radiotherapy, and radiation.

We identified 42 cases reports of malignant priapism secondary to prostate adenocarcinoma, urothelial carcinoma, renal cell carcinoma, rectal adenocarcinoma, caecal carcinoma, oesophageal carcinoma, non-small cell lung cancer, chondrosarcoma, chordoma, melanoma, and lymphoma (Table 1).

**Table 1:** Cases of malignant priapism treated with radiotherapy ^a^RT: radiotherapy for malignant priapism. Ca: carcinoma. NSCLC: non-small cell lung cancer.

Primary Histopathology	No. of cases	No. of cases treated with RT^a^	No. of cases with RT details
Prostate Adenocarcinoma	13	5	2
Bladder urothelial Ca.	9	2	2
Renal cell Ca.	5	2	1
Ureter urothelial Ca.	1	1	0
Rectal adenocarcinoma	3	2	2
Caecal Ca.	2	0	0
Oesophageal Ca.	1	1	1
NSCLC	4	2	1
Chondrosarcoma	1	1	1
Chordoma	1	1	1
Melanoma	1	1	1
Lymphoma	1	0	0
Total	42	16	12

The details of the reviewed case reports are described in Table 2.

**Table 2:** Cases of malignant priapism reviewed NA: information unavailable. Ca: carcinoma. SCC: squamous cell carcinoma.

Primary / Histopathology	Citation	Radiotherapy (Y/N)	Description of radiotherapy technique/dose (Y/N)	Other treatment
Genitourinary tract				
Prostate				
Adenocarcinoma	Mc 1954 [7]	N/A ^a^	N/A	N/A
Adenocarcinoma	Witters, Cornelissen et al. 1985 [8]	N/A	N/A	Palliative
Adenocarcinoma	Osther and Lontoft 1991 [9]	N	N	Hormonal
Adenocarcinoma	Buchholz, Moch et al. 1994 [10]	Y	N	Hormonal
Adenocarcinoma	Schroeder-Printzen, Vosshenrich et al. 1994 [11]	N	N	Palliative, hormonal
Adenocarcinoma	Chan, Begin et al. 1998 [6]	N	N	Hormonal
Adenocarcinoma	Cante, Franco et al. 2014 [12]	Y	Y	Hormonal
Adenocarcinoma	Kitley, Mosier et al. 2010 [13]	Y	Y	Hormonal
Adenocarcinoma	da Silva Gaspar, Nunes et al. 2015 [14]	N	N	Hormonal
Adenocarcinoma	He, Zeng et al. 2012 [15]	N	N	Penectomy, hormonal
Mixed urothelial Ca and adenocarcinoma	Savion, Livne et al. 1987 [16]	Y	N	Hormonal
Adenocarcinoma	Lin, Kim et al. 2011 [2]	N	N	Palliative
Adenocarcinoma	Barrett-Campbell, Petkovska et al. 2018 [17]	Y	N	Chemotherapy, hormonal
Urinary bladder				
Urothelial Ca.	Matthewman, Oliver et al. 1987 [18]	N	N	Chemotherapy
Urothelial Ca.	Kvarstein 1996 [19]	N	N	Palliative
Urothelial Ca.	Chan, Begin et al. 1998 [6]	N	N	Palliative
Urothelial Ca.	Sonmez, Coskun et al. 2009 [20]	Y	Y	No
Urothelial Ca.	Spinapolice, Fuccio et al. 2014 [21]	Y	Y	Palliative
Urothelial Ca.	Ellanti, Connolly et al. 2011 [22]	N	N	Penectomy, chemotherapy
Urothelial Ca.	Al-Mufarrej, Kamel et al. 2006 [23]	N	N	Penectomy
Urothelial Ca.	Zhu, Yao et al. 2012 [24]	N	N	Penectomy
Urothelial Ca.	Ahmed, Elsamra et al. 2012 [25]	N	N	Immunotherapy
Kidney				
Renal cell Ca.	Daniels and Schaeffer 1991 [26]	Y	N	Surgery
Renal cell Ca.	Puppo, Perachino et al. 1992 [27]	N	N	Immunotherapy
Renal cell Ca.	Nezu, Dhir et al. 1998 [28]	Y	Y	No
Renal cell Ca.	Zhu, Xue et al. 2014 [29]	N	N	Palliative
Renal pelvic carcinoma	Liu, Zeng et al. 2014 [30]	N	N	Surgery
Ureter				
Urothelial Ca.	Valadez, Wheeler et al. 1987 [31]	Y	N	No
Gastrointestinal tract				
Rectal adenocarcinoma	Nunes, Matias et al. 2015 [32]	Y	Y	Chemotherapy
Rectal adenocarcinoma	Park, Lee et al. 2009 [33]	Y	Y	Chemotherapy
Rectal adenocarcinoma	Persec, Persec et al. 2014 [34]	N	N	Palliative
Caecal carcinoma	Kapoor, Bera et al. 2012 [35]	N	N	Chemotherapy
Caecal carcinoma	Sasikumar, Harisankar et al. 2013 [36]	N/A	N/A	N/A
Oesophageal carcinoma	Song, Wang et al. 2019 [37]	Y	Y	Chemotherapy
Other				
Lung SCC	Belville and Cohen 1992 [38]	Y	N	No
Lung SCC	Yokoi, Miyazawa et al. 1992 [39]	N	N	Palliative
Lung SCC	Fujimoto, Hiraki et al. 2001 [40]	Y	Y	Radiotherapy to bony metastasis
Lung adenosquamous carcinoma	Guo, Li et al. 2017 [41]	N	N	Penectomy
Chondrosarcoma of the jaw	Cardoso Guimarães, Rodrigues De Souza et al. 2003 [42]	Y	Y	Chemotherapy
Sacrococcygeal chordoma	Mondaini, Mondaini et al. 2005 [43]	Y	Y	Palliative
Melanoma	Sagar and Retsas 1992 [44]	Y	Y	Palliative
Lymphoma	Gong, Zhang et al. 2014 [45]	N	N	Chemotherapy

In 16 cases, palliative radiotherapy was used to treat malignant priapism. The radiotherapy dose fractionation was reported only in 12 cases (Table 3).

**Table 3:** Dose and fractionation of radiotherapy used for the management of malignant priapism NR: not reported. 3D-CRT: 3D conformal radiotherapy. SCC: squamous cell carcinoma.

Primary site & histopathology	Investigator	Radiotherapy technique	Dose and fractionation	Outcome
Prostate adenocarcinoma	Kitley C [13]	NR	40Gy to prostate, base of bladder, and penis	Slow resolution of priapism
Prostate mucinous adenocarcinoma	Cante D [12]	3D-CRT	35Gy in 14 fractions	Complete pain relief and objective response
Bladder urothelial carcinoma	Sonmez NC [20]	NR	56Gy	Partial resolution of priapism
Bladder urothelial carcinoma	Spinapolice E [21]	3D-CRT	30Gy in 10 fractions	Complete pain relief and resolution of priapism
Renal cell carcinoma	Nezu FM [28]	NR	25Gy in 10 fractions	Partial pain relief and decrease in size of erection
Rectal adenocarcinoma	Nunes, B [32]	3D-CRT (one direct anterior electron field)	30Gy in 10 fractions	No acute toxicity; complete response
Rectal adenocarcinoma	Park JC [33]	NR	33.8Gy in 13 fractions	NR
Oesophageal SCC	Song L [37]	3D-CRT	60Gy in 30 fractions	NR
Lung SCC	Fujimoto N [40]	NR	60Gy in 30 fractions	Partial regression of priapism and complete pain relief
Melanoma	Sagar SM [44]	Anterior field	18Gy in 3 fractions, 1 fraction per week	Pain relief and resolution of penile induration
Chondrosarcoma of jaw	Guimaraes GC [42]	NR	25Gy	Partial improvement in pain; decrease in tumor volume
Sacrococcygeal chordoma	Mondaini N [43]	NR	20Gy in 5 fractions	Partial reduction of penile rigidity

Malignant priapism is a rare phenomenon with a poor prognosis [5]. It is unlikely a large prospective study for this condition will be conducted, therefore, management of this condition remains challenging. We reported a case of malignant priapism, secondary to prostate adenocarcinoma, which was successfully treated with high-dose palliative radiotherapy (40Gy in 16 fractions using the VMAT technique). We report that the patient achieved long-lasting symptomatic relief, and the acute toxicity from high-dose palliative radiotherapy was tolerable.

There has been significant improvement in outcomes in patients with metastatic prostate cancer owing to advances in systemic androgen deprivation therapies [46]. However, androgen blockage does not address local symptoms, unlike radiotherapy. There are 13 case reports of malignant priapism secondary to prostate adenocarcinoma, among whom five patients received palliative radiotherapy for priapism symptoms (Table 1). All the patients received concurrent hormonal therapy (Table 2). Unfortunately, only two case reports included brief descriptions of radiation dose fractionation [12-13]. Local symptom control was achieved by administering 35Gy in 14 fractions using the 3D conformal technique [12] to the penis in one case and 40Gy in the other report (fractionation and technique were not described) [13]. Similarly, it has been reported that complete relief pain and priapism can be achieved by locally administering 30Gy in 10 fractions in metastatic bladder urothelial carcinoma [21] and metastatic rectal adenocarcinoma [32] using the 3D conformal technique. On the contrary, priapism symptoms were only partially relieved in earlier case reports [20,28], even with a higher dose (56Gy) [20]. To reconcile the results, it is plausible that the modern conformal radiotherapy technique is better than the field-based technique in terms of ensuring coverage of target and avoiding hotspots or high doses to the organs at risk given the palliative nature of the treatment.

Various doses and fractionations have been reported in the successful treatment of even rarer causes of secondary malignant priapism (Table 3). High-dose radiotherapy (60Gy in 30 fractions) partially relieved priapism symptoms in non-small cell lung cancer, but the acute toxicities were not described in detail [40]. Hypo-fractionated radiotherapy is effective in control priapism secondary to melanoma [44] but not soft tissue neoplasm [42-43]. In sum, these case reports demonstrate that high-dose palliative external beam radiotherapy is an effective palliative option for priapism. However, this option has been relatively underutilized. Only 16 out of 42 malignant priapism cases were reported to be treated with palliative radiotherapy according to our review.

One reason that palliative radiotherapy may be underutilized is that the traditional patient setup is challenging. In the present case, it was very difficult for the patient to tolerate the initial treatment setup, which used a traditional plastic block or wax to hold the penis, due to significant pain. Furthermore, the patient had previously received high-dose radiotherapy to the pelvis. In this case, we used the VMAT technique to overcome these issues. The VMAT setup is simple and comfortable for the patient. Our setup is also reproducible, as we use imaging guidance.

To summarize, the paucity of literature regarding the successful treatment of malignant priapism via radiotherapy means that the optimal radiation dose and fractionation to use is unknown. We recommend high-dose palliative radiotherapy (40-50Gy) for malignant priapism secondary to prostate cancer to achieve durable local control. This dosage was shown to be effective in the current case report and a further two other reports [12-13]. This dose may also be effective for other common causes of malignant priapism (e.g. urothelial carcinoma or rectal carcinoma). The VMAT radiotherapy technique is preferred for two reasons. First, it allows for a simple patient setup and avoids additional stress to the patient. Secondly, malignant priapism is primarily caused by genitourinary or lower gastrointestinal malignancies. Patients with genitourinary or lower gastrointestinal malignancies typically receive high-dose radiotherapy as part of their initial treatment or may require further palliative pelvic radiotherapy. VMAT helps minimize damage to normal pelvic tissues caused by retreatment of radiotherapy.

## Conclusions

Malignant priapism is a rare clinical presentation in patients with metastatic carcinoma. High-dose palliative radiotherapy is an effective treatment for symptomatic relief. Patients need to be monitored closely during treatment to avoid excessive toxicity from radiotherapy.
